# Screening of Aptamers on Microfluidic Systems for Clinical Applications

**DOI:** 10.3390/s120709514

**Published:** 2012-07-11

**Authors:** Chen-Hsun Weng, Chao-Jyun Huang, Gwo-Bin Lee

**Affiliations:** 1 Department of Microbiology and Immunology, National Cheng Kung University, Tainan 70101, Taiwan; E-Mail: b88501113@gmail.com; 2 Department of Mechanical Engineering, National Taiwan University, Taipei 10617, Taiwan; E-Mail: c.j.huang0706@gmail.com; 3 Department of Power Mechanical Engineering, National Tsing Hua University, Hsinchu 30013, Taiwan

**Keywords:** microfluidics, SELEX, aptamer, biosensor, MEMS

## Abstract

The use of microfluidic systems for screening of aptamers and their biomedical applications are reviewed in this paper. Aptamers with different nucleic acid sequences have been extensively studied and the results demonstrated a strong binding affinity to target molecules such that they can be used as promising candidate biomarkers for diagnosis and therapeutics. Recently, the aptamer screening protocol has been conducted with microfluidic-based devices. Furthermore, aptamer affinity screening by a microfluidic-based method has demonstrated remarkable advantages over competing traditional methods. In this paper, we first reviewed microfluidic systems which demonstrated efficient and rapid screening of a specific aptamer. Then, the clinical applications of screened aptamers, also performed by microfluidic systems, are further reviewed. These automated microfluidic systems can provide advantages over their conventional counterparts including more compactness, faster analysis, less sample/reagent consumption and automation. An aptamer-based compact microfluidic system for diagnosis may even lead to a point-of-care device. The use of microfluidic systems for aptamer screening and diagnosis is expected to continue growing in the near future and may make a substantial impact on biomedical applications.

## 1. Introduction

SELEX (systematic evolution of ligands by exponential enrichment) is a method to screen single-stranded DNA (ssDNA) or RNA ligands from a random library of nucleotide sequences [1]. The ligands that are selected via SELEX are called aptamers [1–3]. Aptamers have many advantages compared to antibodies. For instance, they can be produced easily and inexpensively, and aptamers are simple to modify chemically and to integrate into different analytical methods [1–3]. Moreover, aptamers have a strong affinity and a high specificity to the target molecule and can be labeled with different functional groups [4]. SELEX research was first reported in the 1990s by Gold and Ellington [1,3], and a typical process is as follows: first, a combinatorial nucleic acid library (ssDNA or RNA) is synthesized. The sequence of oligonucleotides in the library is composed of random sequences in the middle and flanked by fixed sequences as primer binding sites. The length of the random region is normally between 20 to 40 base-pairs, which create a library with a large number of random sequences (10^15^ to 10^16^) [3–5]. The library is then incubated with the desired target molecule for binding. Next, the unbound nucleic acids are washed away from those bound specifically to the target molecule, which are then eluted from the target molecule and amplified by a polymerase chain reaction (PCR). This selection procedure is repeated for several rounds until the resulting sequences are highly enriched. The selected nucleic acids are subjected to sequencing and synthesis to test for their potential binding affinity. The SELEX technology generates aptamers with a high binding affinity and specificity. These advantages have made them very promising in analytical, diagnostic and therapeutic applications [6–9]. Aptamers are short single-stranded nucleic acid oligomers with a specific and complex three-dimensional structure [10]. Based on their three-dimensional structures, aptamers can bind well to a wide variety of targets. Binding of the aptamer to the target is due to structural compatibility, electrostatic interactions, van der Waals interactions, and hydrogen bonding [11].

Since the discovery of aptamers, many researchers have used the SELEX process to select aptamers with high affinities and specificities for their targets [12–15]. Many of the selected aptamers show affinities comparable to those observed for antibodies. Recently, researchers have moved to a microfluidic chip/system to perform SELEX that can be optimized, giving significant advantages in terms of increased speed and reduced costs [16–20]. Furthermore, such microfluidic chips/systems would be a candidate for the high-throughput applications. At the heart of the microfabrication process is the generation of precisely defined wells, mixers, valves and pumps onto silicon, glass or polymeric substrates. Various examples of self-contained, fully integrated, miniaturized devices will be reviewed in the following sections.

## 2. Screening of Aptamers on Microfluidic Chips

Typically, the SELEX method is an iterative process of incubation, separation, and nucleic acid amplification. Multiple rounds of selection are generally necessary to screen aptamers with a sufficient specificity and a high binding affinity, which requires more sample/reagent consumption and time [21]. In order to accelerate this lengthy screening process, a wide variety of microfluidic incubation, separation and amplification techniques have been explored as means to enhance the efficiency of aptamer selection, including capillary electrophoresis (CE), sol-gel isolation and magnetic-bead-based selection have been reported in literature [22–24]. To address the need for a method to rapidly, efficiently, cost-effectively, and reproducibly select high affinity ligands, we will review herein various developed microfluidic devices. These chips are usually low cost, easily reproducible and may be disposable. Furthermore, the labor-intensive process may be shortened due to the automation enabled by microfluidic technologies.

### 2.1. CE Microfluidic Chips for Screening of Aptamers

**Figure 1.**
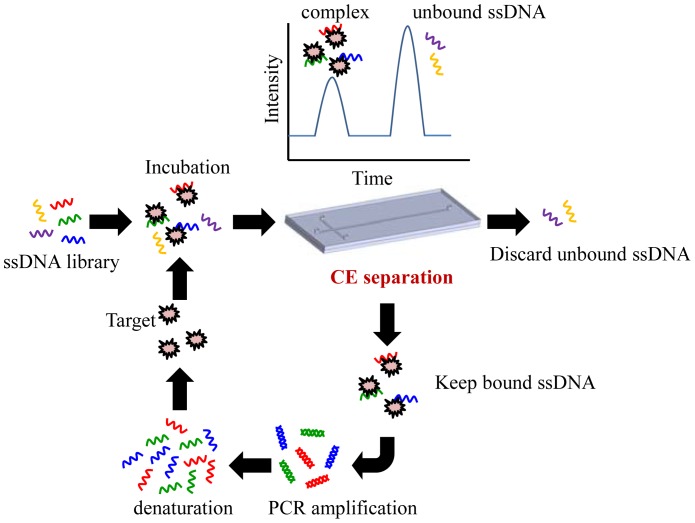
Schematic illustration of the SELEX processes in CE-microfluidic chips. A library of ssDNA is incubated with the target molecules. Capillary electrophoresis is used to separate bound sequences. Binding nuclear acids are amplified by PCR and purified giving an enriched ssDNA pool which suitable for further rounds of selection. High-affinity aptamers are typically obtained after two to four rounds of selection.

Recently, some research groups have demonstrated CE microfluidic chips as an efficient SELEX selection method (CE-SELEX) (see Figure 1) [22,25–27]. In CE-SELEX, the random ssDNA library is first incubated with the target in a solution. The mixture is then injected into a CE chip and separated electrokinetically under a high voltage. CE-SELEX utilizes electrophoresis to separate binding sequences from inactive sequences by a mobility shift to allow separation. Nucleic acids that are bound to the target migrate with a different mobility from the unbound sequences, and then the selected nucleic acids could be collected afterwards. Bound nucleic acids are then amplified and purified off-chip for multiple rounds of selection, similar to the protocol in a conventional SELEX. CE-SELEX has been used to successfully isolate aptamers for large targets such as human immunoglobulin E [25] and protein kinase C [26]. More recently, experimental data has shown that CE-SELEX could also be used to isolate aptamers that are bound to smaller targets such as neuropeptide Y [28]. The unique advantage of the CE-SELEX method is the ability to rapidly obtain a specific aptamer. Furthermore, the high partitioning efficiency of the CE-SELEX method in comparison with the traditional SELEX method decreases the number of rounds of SELEX to 1–3 rounds. In general, the incubation time of the CE-SELEX is less than an hour at room temperature. The short incubation time also maintains the activity of the targets.

In particular, the CE-SELEX method has shown excellent selection efficiencies for protein targets that exhibit significant shifts in the electrophoretic mobility; protein-binding aptamers in the low-nanomolar range have been isolated after a small number of selection rounds [22,25]. However, the method is less effective for other classes of targets, like cell surfaces. Moreover, the nanoliter volume of a random library is a limitation of CE-SELEX. This small volume limits the number of library sequences that can be assessed and requires a highly concentrated library. CE-SELEX has already been adopted by several research groups to identify aptamers for a number of targets [25–28]. Aptamers with low nanomolar to high picomolar dissociation constants have been identified. These dissociation constants are similar to those of antibodies identified using conventional protocols. CE-SELEX is performed in a free solution, thus reducing the non-specific interactions and excluding immobilization strategies. The high resolution capability of CE chips increases the rate of enrichment allowing for high affinity aptamers to be obtained within 2–4 rounds of selection. However, the CE-SELEX method still needs to be integrated with a PCR amplification module in order to automate the entire microfluidic platform. However, this method still has some practical limitations. For instance, when the target is an extremely small molecule, the velocity of nucleic acids that are bound to the target is close to that of the unbounded nucleic acids; thus, it is difficult for CE-SELEX to separate the unbound nuclear acids. Another disadvantage of this method is that the whole SELEX protocol has not been automated. In summary, using the CE-SELEX process to separate unbound oligonucleotides is a crucial step for successful aptamer selection. There is still a great need for more integration with other microfluidic devices to form a fully-automated system.

### 2.2. Sol-Gel Microfluidic Chips for Screening of Aptamers

**Figure 2.**
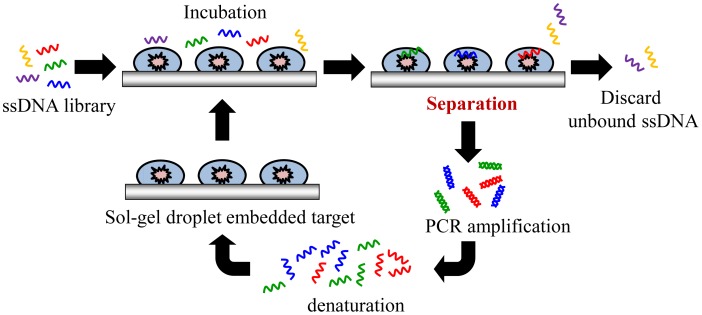
Schematic illustration of the SELEX processes in sol-gel microfluidic chips. A library of ssDNA is incubated with sol-gel arrays of proteins in a microfluidic system for efficient selection of ssDNA aptamers against target molecules. The sol-gel microfluidic chips greatly improved selection efficiency, reducing the number of selection cycles needed to produce high affinity aptamers.

Sol-gel entrapment of biomolecules, such as proteins, has been used as a promising selection method of considerable interest to researchers [23]. For instance, a nanoporous composite made by the sol-gel process provides an aqueous environment that maintains biological activity and enhances biomolecule stability. Recently, some research groups have reported on a screening strategy by using a sol-gel microfluidic chip to immobilize various targets [19,23,29,30]. Sol-gels are synthesized using silicate glasses with nanoporous structures. They have an ability to entrap proteins and maintain their activity over months. Protein immobilization in sol-gels can hold a protein without requiring affinity capture tags, thus enabling the entrapment of various proteins in their native state [23]. Sol-gels have been used to successfully immobilize targets such as recombinant yeast TATA binding protein and yeast transcription factor IIB protein [30]. Furthermore, a new sol-gel system with integrated microheaters to perform the SELEX process has been reported, as shown in Figure 2. This sol-gel system can hold large amounts of active proteins in a nanoporous sol-gel [19]. The sol-gel microfluidic system allows for sufficient binding reactions between the library pools and the immobilized target proteins. Thus, a sol-gel SELEX chip can isolate aptamers onto target proteins. Furthermore, high-affinity aptamers for the specific proteins can be selectively screened after multiple cycles of the SELEX. Next, microheaters could be further incorporated in the microfluidic chip for performing the thermal elution, which has shown higher elution efficiency than other elution methods like elution by ionic strength [30]. However, the microheater has not been used for an on-chip PCR process, and therefore an off-chip PCR process is inevitable. Moreover, this system needed syringe pumps to transport the reagent. Furthermore, in order to form a sol-gel microfluidic device, the sol-gel manufacturing process is relatively complex [30]. Nonetheless, a sol-gel microfluidic system allows for sufficient binding reactions between nucleic acids and target proteins. Thus, it can lead to the isolation of aptamers specific to many of the target proteins, and improve the selection of aptamers to these specific proteins.

### 2.3. Magnetic-Bead-Based Microfluidic Chips for Screening of Aptamers

**Figure 3.**
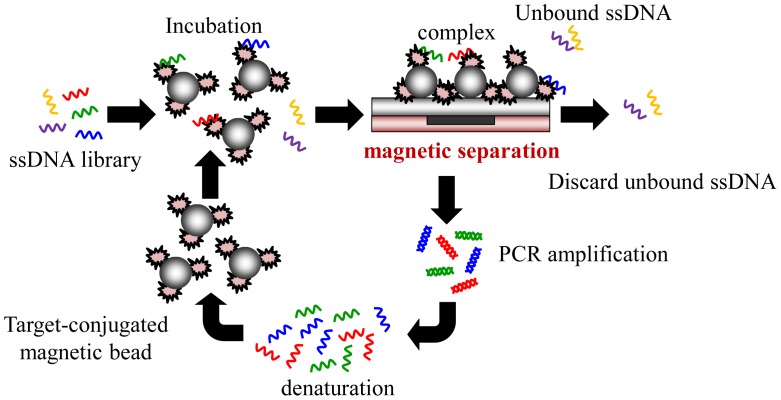
Schematic illustration of the SELEX processes in magnetic-bead-based microfluidic chips. The microfluidic selection process begins with the incubation of random ssDNA library with target proteins conjugated to magnetic beads. After incubation, the partitioning step to separate the target-bound aptamers from the unbound nuclear acids is performed in the microfluidic chip. Stringent washing conditions then are imposed in the microchannel to continuously elute weakly- and unbound nuclear acids from the microfluidic chip. After the separation, the external magnets are removed, and the beads carrying the selected aptamers are released from the device. The entire separation process with trapping, washing, and bead elution performs on chip. Finally, the selected aptamers are amplified via PCR.

Recently, some research groups reported the application of microfluidic technologies for SELEX utilizing magnetic separation in a continuous-flow, magnetically-activated, chip-based separation device [17,20,21,24], as shown in Figure 3. However, the ferromagnetic structures within the microchannel are complex to microfabricate. Also, this method must precisely generate magnetic field gradients to capture the magnetic beads. Using this method, some groups have demonstrated the isolation of DNA aptamers against the light chain of recombinant botulinum neurotoxin type A and streptavidin [20,21]. In addition to protein molecules, it is believed that the magnetic-bead-based selection approach may be readily extended for use with cells toward the rapid generation of aptamers as cell-surface markers. The magnetic-bead-based SELEX is advantageous since it can be used for any bead-bound target and can be isolated by simply applying a magnetic field. However, most magnetic-bead-based microfluidic devices are not integrated with micropumps, microvalves, or an on-chip PCR module and, therefore, hinder their practical application.

**Figure 4.**
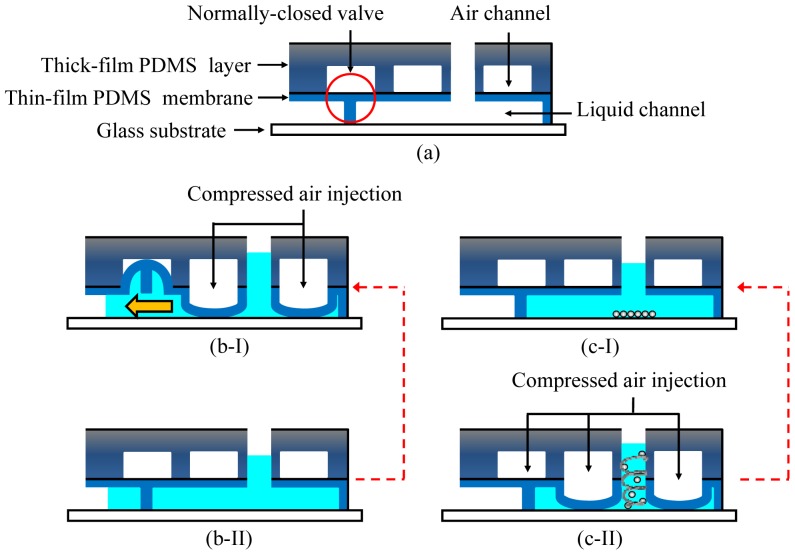
(**a**) Schematic illustration of the pneumatic micropump/micromixer; (**b**-I) The working principle of the membrane-type micropump. The forward-motion of the fluids in the microchannel is activated as the compressed air is injected; (**b**-II) The fluid in the microchannel is driven from the right side to the left side; (**c**-I) Initial state of the micromixer without a supply of compressed air; (**c**-II) The mixing state of the micromixer when supplied with compressed air.

Recently, integrated microfluidic systems using magnetic beads to provide an automated approach to rapidly screen aptamers has been reported [31,32]. Furthermore, magnetic bead-based selection methods have been shown to be effective for any class of molecular targets that can be immobilized onto the bead surfaces, including small molecules, proteins, and cell surfaces. The screening process incubates magnetic beads conjugated with a target in a random nucleic acid library to find the specific aptamers. Then, any bead-bound nucleic acids can be captured and purified by applying a magnetic field. This aptamer extraction process can even be further performed in a microchannel. The use of magnetic beads to select aptamers in a microchannel has improved the efficiency of the SELEX method [31,32]. However, these microchannel chips can only be applied to the extraction process, not to the entire iterative SELEX process. The critical incubation step for ssDNA to bind to the magnetic beads and the PCR process still are time-consuming and labor-intensive. Recently, a microfluidic chip which integrated an incubation module with an on-chip nucleic acid amplification module for the rapid screening of aptamers has been reported [31,32]. Several important components such as micropumps, microvalves, micromixers and temperature control system can be integrated to perform the entire iterative SELEX process. In these studies, the developed micropump can be also used as a micromixer when compressed air is supplied to the normally-closed microvalve as shown in Figure 4(a). The working principles for the micropump and the micromixer are schematically illustrated in Figure 4(b,c), respectively. Not only do these components simplify the control scheme, but they also prevent cross contamination during the screening process. Furthermore, the consumption of samples and reagents and the screening time for SELEX process are significantly reduced when compared with the channel-based microfluidic chips.

Using a multi-function microfluidic chip, the aptamer specific to alpha-fetoprotein (AFP) and C-reactive protein (CRP) were successfully screened. Using a multi-function microfluidic chip, the aptamer specific to alpha-fetoprotein (AFP) and C-reactive protein (CRP) were successfully screened. It is envisioned that the fully-automated SELEX process on an integrated microfluidic system may provide a user-friendly, efficient and rapid platform for screening aptamers for a variety of biomedical applications.

Another issue involved in the aptamer screening process is that it requires a time-consuming competitive assay to select ssDNA with a high affinity and specificity for subsequent sequencing and synthesis [32,33]. This is because only a small amount of the screened ssDNA can be used for further applications. Therefore, a competitive assay chip is necessary to screen for the high affinity and specificity ssDNA after the SELEX selection process. In order to address these problems, a magnetic bead-based microfluidic system which consisted of a new micro-injector for transport of the PCR reagent, and a new array-type microheater to improve the thermal uniformity inside the PCR reaction area which enhances the entire SELEX process, has been reported recently [32]. More importantly, the screened ssDNA sequences were determined to have a high affinity and high specificity to the target molecules by using a new competitive assay chip. The competitive assay device could efficiently remove weakly bound and non-specific ssDNA. With this approach, the cost of ssDNA sequencing and synthesis can be greatly reduced. Experimental results showed that an aptamer specific to AFP and CRP were successfully screened [31,32].

## 3. Cell-SELEX in Microfluidic Chips

**Figure 5.**
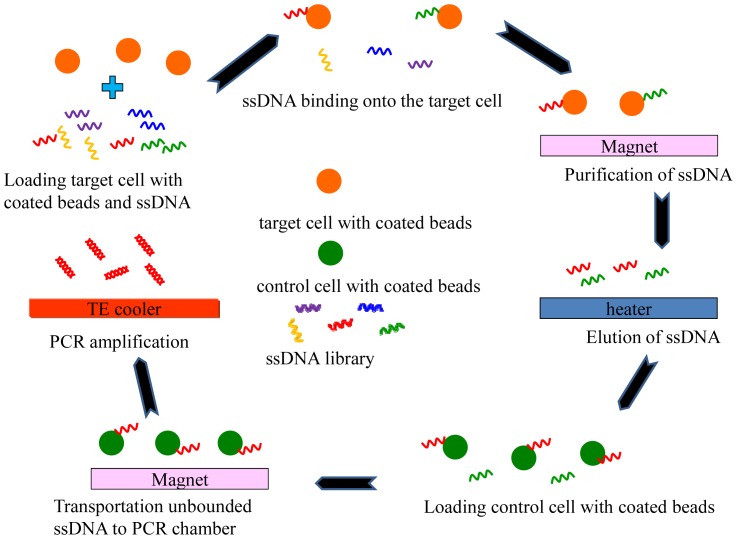
Schematic illustration of the Cell-SELEX processes in magnetic-bead-based microfluidic chips. Aptamers were bound to the target cells, and control cells were used for negative counter-selection. After 15–20 cycles of positive/negative selection, the highly-specific aptamer for the target cells can be identified.

A modification of the traditional SELEX process that uses cells as targets has been named Cell-SELEX [34–36]. Cell-SELEX is a method to screen for aptamers from a combinatorial nucleotide library. The aptamer with a high affinity for the target cells are screened and those with an affinity for the control cells are filtered out (negative selection). The Cell-SELEX process requires multiple rounds (typically 15–25 rounds) of extraction and PCR amplification which needs a significant number of cells and is therefore a time-consuming, labor-intensive process. After screening, for example, aptamers with a high affinity and specificity for cancer cells can be identified. This makes aptamers an attractive tool for cancer research and biomarker discovery [37,38]. The Cell-SELEX process can be also implemented in a microfluidic system that uses magnetic beads to capture the target aptamers to greatly improve the affinity and specificity. However, the traditional Cell-SELEX is very time-consuming. Thus the activity of cells cannot be maintained and, therefore, different cells may be tested in different rounds. Recently, an automatic, magnetic bead-based microfluidic system which integrated an extraction device and an on-chip PCR for the fast screening of aptamers has been demonstrated [39], as shown in Figure 5. The entire Cell-SELEX process was performed on a single chip within a shorter time when compared to traditional Cell-SELEX protocols. Thus, Cell-SELEX brings a promising approach to accelerate the progress of biomarker discovery. In order to perform the Cell-SELEX on a single microfluidic chip, three modules, including a microfluidic control module, a magnetic bead-based aptamer extraction module, and a temperature control module for cooling reagents and fast nucleic acid amplification have been integrated [39]. In addition, identification of potential biomarkers by using cell-binding aptamers was addressed. It is believed that using the Cell-SELEX strategy on microfluidic systems represents a significant improvement over the existing methods for biomarker identification. It allows for automated screening of cell-specific aptamers, which the conventional SELEX cannot achieve. The magnetic bead-based SELEX could have broader uses than its counterparts. The magnetic bead-based microfluidic system could be further used for diverse in-vitro selection techniques such as phage display, mRNA display, or a ribosome display library.

## 4. Aptamer Assays for Clinical Applications

**Figure 6.**
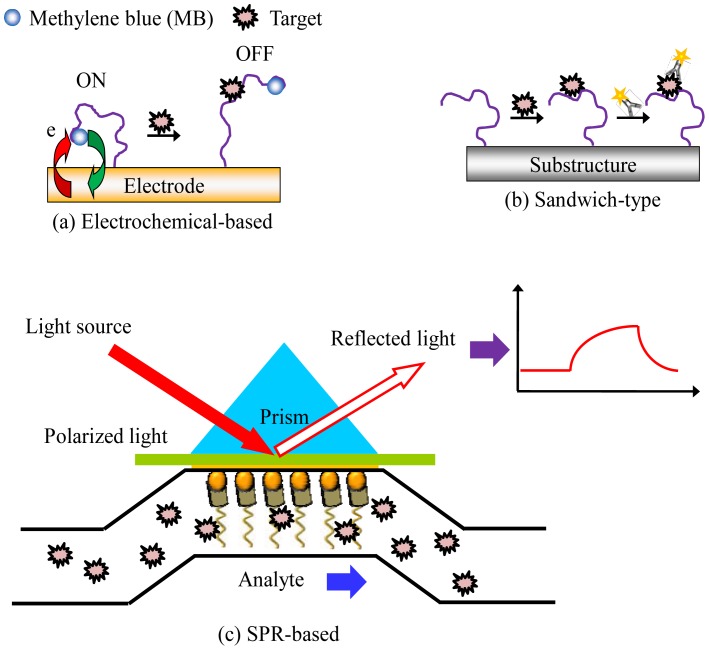
Illustrations of various detection techniques for aptamer-based assays in microfluidic chips including (**a**) an electrochemical-based detection: a simple and sensitive method has been developed, the microchannel is immobilized aptamer. Then, the target proteins are sequentially captured by the specific aptamer–protein interaction. Finally, an electrochemical system is employed to detect the current response; (**b**) a fluorescent-based detection method: the sandwich assay, which is one of the most used assay formats. In this approach, the analyte is sandwiched by two specific probes, one capture probe and the other reporter probe. Capture probes as aptamers are often immobilized on microchannels, while reporter probes as antibodies are often conjugated with signaling moieties (e.g., fluorescent, enzymes or nanoparticles (NPs)); (**c**) a SPR sensing scheme: In this SPR sensing device, a selective surface is formed by immobilizing the aptamer on the surface. Then, the target is injected at a constant flow rate, while the instrument measures changes in the resonance angle that occur at the surface. The angle changes due to aptamer binding to the target. The signal is proportional to the number of bound molecules, thus the SPR method allows for label-free detection in a microfluidic chip with single-site binding resolution.

Another interesting area of aptamer research is their applications in clinical diagnostics and biosensing. Their high affinity, high specificity and ease of modification make aptamers excellent diagnostic or biosensing tools. For aptamer-based diagnostics or biosensors, binding of an aptamer to its target molecule have been extensively explored [40–42]. Detection techniques including electrochemical detection [43–46], fluorescent and colorimetric detection [47,48], surface plasmon resonance (SPR) sensing [49,50], and many other detection schemes can be combined with microfluidic chips for aptamer-based biomedical applications, as shown in Figure 6.

For example, a microfluidic-based, aptamer-sandwich-type assay using electrochemical detection of human thrombin has been reported [43]. Thrombin is a type of serine protease which has a high prelevance in some pathological conditions [51]. In this work, the microfluidic aptamer-based sensor exhibited a low detection limit (1 pM) and could detect thrombin in a human serum sample because the aptamer had a high specificity to human thrombin. Thus, this microfluidic electrochemical-based aptamer sensor has the potential for detecting the target molecule in a complex mixture (see Figure 5(a)).

Another excellent work using a highly specific and sensitive DNA aptamer, instead of using antibodies, for the brain natriuretic peptide (BNP) assay has been reported by using Agilent lab-on-a-chip (Agilent Technologies, Waldbronn, Germany) technology [52]. BNP is a neurohormone that causes diuresis, natriuresis and vasodilatation. The detection of the BNP concentration has practical applications in emergency medicine. Therein, the Agilent system integrated with the fluorescently-labeled aptamer improves the sensitivity for clinical BNP detection. It also improves detection performance and speed, and reduces the cost and reagent consumption involved in the detection of BNP.

Alternatively, SPR is an optical technique that detects refractive index changes and has been extensively applied to bio-analysis [50]. SPR allows a large matrix of samples to be simultaneously measured by monitoring the fixed angle reflectivity change due to sample binding [49]. DNA, RNA, and protein interactions in arrays have been analyzed with a high sensitivity by SPR. SPR has also been integrated with a microfluidic device [50]because the microfluidic system can provide a uniform velocity profile across the sample matrix surface to make the results reproducible (see Figure 5(b)). It also provides an automatic sampling platform for SPR sensing. A microfluidic system was used to minimize the mass transfer limitations and to enable kinetic analysis. The high quality of the detection results demonstrated the advantage of this method using integrated microfluidic techniques.

Another aptamer-based assay reported a microfluidic device capable of aptamer–protein specific binding for the determination of CRP by using an optical detection scheme [53]. Herein, a microfluidic system to automatically perform the entire protocol for CRP detection with a small sample volume has been demonstrated. This microfluidic system was composed of pneumatically-driven micropumps, normally-closed microvalves, and a vortex-type micromixer. Furthermore, a new micro-injector was developed to automate the entire detection process. The micro-injector had a unique feature that enabled rapid injection and was integrated with the pneumatic micropump to perform the washing and developing processes. The aptamer was conjugated on the surface of the magnetic beads for recognition of CRP in the serum. Then, a custom-made optical detection system has also been developed to measure the concentration of the CRP in human serum. The developed microfluidic system has a low limit of detection and can perform the entire detection protocol within 30 min [53]. Moreover, the AFP-specific aptamer was validated with an AFP immunoassay chip using a similar approach [32]. The detection limit for AFP was experimentally found to be 12.5 ng/mL. Therefore, the developed immunoassay chip could be used to measure AFP concentrations within a reasonable linear range.

Aptamers have a better applicability than antibodies in microfluidic systems because they are more easily labeled with fluorescent tags and more stable for longer periods of time under various conditions. Recently, aptamers have been used as excellent affinity probes in microfluidic chips using different methods. For instance, electrophoretic separation and detection of aptamer-protein complexes for the analysis of thrombin in plasma has been reported [54]. An aptamer-based, miniaturized, affinity chromatograph has also been developed by immobilizing photo-cleavable RNA aptamers onto magnetic beads [55]. The hepatitis C virus RNA polymerase was successfully detected at an extremely low concentration of 9.6 fmol [55]. This microfluidic system with a miniaturized, high-throughput, affinity chromatograph chip can detect and identify various molecules such as antigens and pathogens. Recently, an aptamer-based sandwich assay for thrombin detection employing magnetic beads and quantum dots on microfluidic chips has been reported [56]. This microfluidic chip facilitated the aptamer sandwich assays for the determination of thrombin with a higher specificity than direct immunoassays. Thus, aptamer-functionalized magnetic beads were first used to capture thrombin in microfluidic chips and another different aptamer functionalized with quantum dots was also employed for microfluidic detection of thrombin. The binding of thrombin to the two different aptamers was via the sandwich assay. Since the secondary aptamer was labeled with quantum dots, thrombin could be measured by fluorescence microscopy. This method enabled rapid thrombin detection with a high specificity and the limit of detection was 10 ng/mL [56].

A microchip-based, aptamer-sandwich-type sensor probe has the potential for screening different levels of biomarkers in a complex sample. One of the most challenging targets in aptamer-based assays is cancer cells. Aptamer-coated microchannels for detecting cancer cells have been recently demonstrated [57]. A microfluidic chip that can sort, enrich, and then detect multiple types of cancer cells from a complex sample was successfully reported. It implemented cell-affinity chromatography based on the selective cell-capture of immobilized aptamers. This enrichment can be achieved because the height of the channel is on the order of a cell diameter. Thus, by using the aptamer-based device, cell capture was achieved simply and inexpensively, with no sample pretreatment before cell analysis. Enrichment and simultaneous detection of multiple types of rare cancer cells can be further used to detect early stage cancers.

Finally, the selection of aptamers for specific subtypes of circulating tumor cells and stem cells (or cancer stem cells) using microfluidic systems that can perform extraction, enrichment, multiplexing, sorting, and separation poses great promise. Thus, counting rare cancer cells can be used to diagnose metastatic relapse, monitor response to drugs and therapies, and to track tumor progression. Cell-SELEX on microfluidic systems may play an important role in the development of these promising detection platforms. Table 1 lists the processes for aptamers screening and detection in microfluidic chips, including their advantages and disadvantages. For instance, the sol-gel microfluidic chips entrap various proteins by their native state. They can hold a protein without requiring affinity-capture tags. The disadvantages of sol-gel microfluidic chips for screening of aptamers include relatively time-consuming and higher production cost. Alternatively, the SPR technique has the advantages of label-free, real-time detection and high throughput for bio-molecular interaction analysis. However, the SPR measures the mass of materials binding onto the sensor surface, very limited analytes (molecular weight <1000 Da) only produce very small response. Thus only under optimal conditions, the signal-to-noise ratio could be satisfactory. However, the CE technique has also some limitation. When the target is a small molecule, the velocity of the nucleic acid-target complex is close to the nucleic acid itself, the separation of the nucleic acid-target complex and the nucleic acid cannot work well by using the CE chip method. Alternatively, the magnetic-bead-based microfluidic chip was demonstrated to be rapid, highly efficient, automatic and applicable to a wide range of targets.

Different microfluidic systems for the SELEX technology have been applied for different kinds of clinical targets. Inorganic and small organic molecules, proteins, as well as complex targets like target mixtures or whole cells were used for SELEX. In this review, several publications summarized the various target molecules according to different microfluidic chips. Human immunoglobulin E [25], protein kinase C [26], neuropeptide Y [28], recombinant yeast TATA binding protein [30], yeast transcription factor IIB protein [30], the light chain of recombinant botulinum neurotoxin type A [20], streptavidin [21], alpha-fetoprotein [31], C-reactive protein and cancer cell [32] are important in clinical applications. The screened specific aptamers can be used for clinical diagnostic and therapeutic applications.

## 5. Conclusions

In this review article, recent works on automating aptamer selection using microfluidic devices have been extensively reviewed. Microfluidic systems for sample pretreatment to purify the specific samples in an aptamer-based assay have also been reviewed and discussed. Even with the advances in these microfluidic systems, there are several issues that were identified and need to be resolved before they can be used for clinical applications. However, based on the selected aptamers, applications including clinical diagnostics and biosensing for the identification of various target molecules can be realized. For each of these applications, the initial choice of enabling technology and detection strategy is important to select an aptamer and/or target molecule with optimal binding properties. Moreover, the microfluidic technology has many advantages over conventional counterparts including less sample/reagent consumption, lower power consumption, less contamination, and automation. Microfluidic devices and systems have extensively used aptamers for a variety of biomedical applications such as cell-based assays, molecular diagnosis, immunoassays, and biochemical assays. It is expected that these microfluidic devices and systems may soon provide a fast and accurate clinical analysis with an aptamer-based sensor. Most importantly, point-of-care devices and analysis systems may eventually be feasible in the near future.

## Figures and Tables

**Table 1.** Summary for screening methods and detection methods in microfluidic chips.

**Screening Methods**	**Principles**	**Advantages**	**Disadvantages**
CE method	Electrophoretic separation is performed to separate binding sequences from inactive sequences by a mobility shift.	Rapid process, high partitioning efficiency	The number of library sequences is relatively smaller, not fully-automated, and difficult to separate the extremely small molecule. It also requires an off-chip PCR process.
Sol-gel method	Sol-gels entrap various proteins by their native state.	Sol-gels can hold a protein without requiring affinity-capture tags.	Relatively time-consuming fabrication process and higher production cost.
Magnetic-bead-based method	Magnetic separation is performed in a continuous-flow, magnetically-activated, chip-based separation device.	It can be used for any bead-bound target and can be isolated by simply applying a magnetic field. It can be fully automated if integrated with other microfluidic control devices	Bead aggregations may cause blockage in the microchannel.
**Detection Methods**	**Principles**	**Advantages**	**Disadvantages**

electrochemical detection	Electrochemical method is employed to detect the current or voltage signals.	High sensitivity, fast response, low cost, easy fabrication, and the possibility for miniaturization	Unsatisfactory limit of detection
fluorescent-based detection	The analyte is sandwiched by two specific probes, one capture probe and another reporter probe.	High detection performance, fast analysis, satisfactory detection limit	Fluorescent labeling, relatively expensive equipment
SPR methods	The analyte is measured by monitoring the fixed angle reflectivity change due to sample binding.	Label-free, real-time and high-throughput	Mass transport can affect kinetic analysis; relatively expensive equipment.
